# Two challenging cases of anti-MDA-5 dermatomyositis with rapidly progressive interstitial lung disease

**DOI:** 10.1093/omcr/omae061

**Published:** 2024-06-07

**Authors:** Kathryn Biddle, Elif Agaoglu, Geoffrey Brent, John Adam, Rachel Nockels, Adam Loveridge, Farid Bazari, Janakan Natkunarajah, Israa Al-Shakarchi

**Affiliations:** St George's University of London, Infection and Immunity, Cranmer Terrace, London, SW17 0RE, UK; Kingston Hospital NHS Foundation Trust, Rheumatology, Kingston upon Thames, KT2 7QB, UK; Kingston Hospital NHS Foundation Trust, Respiratory, Kingston upon Thames, KT2 7QB, UK; Kingston Hospital NHS Foundation Trust, Dermatology, Kingston upon Thames, KT2 7QB, UK; Kingston Hospital NHS Foundation Trust, General Medicine, Kingston upon Thames, KT2 7QB, UK; Kingston Hospital NHS Foundation Trust, General Medicine, Kingston upon Thames, KT2 7QB, UK; Kingston Hospital NHS Foundation Trust, Respiratory, Kingston upon Thames, KT2 7QB, UK; Kingston Hospital NHS Foundation Trust, Respiratory, Kingston upon Thames, KT2 7QB, UK; Kingston Hospital NHS Foundation Trust, Dermatology, Kingston upon Thames, KT2 7QB, UK; Kingston Hospital NHS Foundation Trust, Rheumatology, Kingston upon Thames, KT2 7QB, UK

**Keywords:** dermatology, rheumatology, respiratory disorders, immunology, critical care medicine

## Abstract

Anti-MDA-5 dermatomyositis (DM) is a subtype of idiopathic inflammatory myopathy, commonly presenting as clinically amyopathic dermatomyositis. It is associated with rapidly progressive interstitial lung disease and a poor prognosis. Here, we present two cases of anti-MDA-5 DM and discuss the challenges associated with timely diagnosis, and the importance of early and aggressive treatment.

## Introduction

Idiopathic inflammatory myopathies (IIM) are systemic autoimmune conditions with varying clinical manifestations. Myositis-specific autoantibodies (MSA) are detected in over 50% of IIM cases and characterise distinct clinical phenotypes [1, 2].

Anti-MDA-5 dermatomyositis (DM) is defined by the presence of autoantibodies against melanoma differentiation-associated protein 5 (MDA-5) [3]. It is a subtype of IIM, representing 1.3%–10% of cases in Europe [2] and 15%–36% of cases in Chinese cohorts [4]. Anti-MDA-5 DM was first described in East-Asian patients with cutaneous manifestations of DM with mild or no muscle involvement (hypomyopathic DM or clinically amyopathic DM (CADM)) [5]. Due to its association with rapidly progressive interstitial lung disease (RP-ILD), anti-MDA-5 DM carries a poor prognosis [2].

This report summarises two cases of anti-MDA-5 DM, presenting in short succession to a district general hospital. Throughout the report, we will discuss the challenges associated with timely diagnosis and the importance of prompt treatment.

## Case reports

### Case A

A 47 year-old female of Egyptian heritage with no medical history, presented to acute medicine. She reported three weeks of erythema of her fingers, and a maculopapular rash overlying her elbows and thighs. She also described a 10-day history of early morning stiffness with joint pain and swelling, and respiratory symptoms, including dry cough and shortness of breath on exertion.

**Figure 1:**
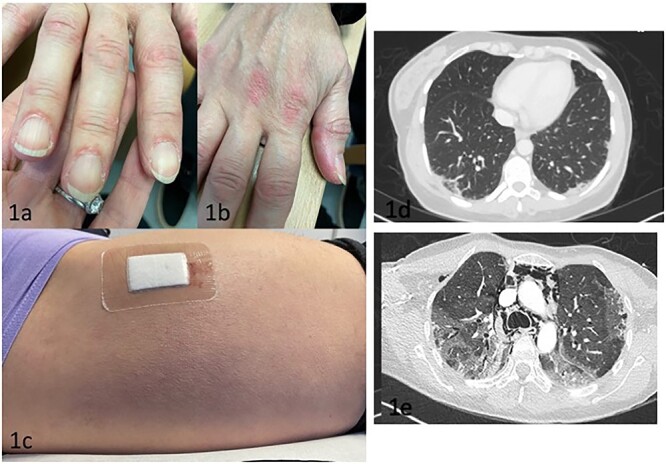
Images of the skin and CT changes seen in case A (a) Right hand digits showing periungual swelling and erythema (b) Gottron’s papules on the right second and third metacarpophalangeal joints. Rough and cracked skin can be seen on the lateral aspect on the thumb, consistent with mechanic’s hands. (c) Right lateral thigh showing a diffuse erythematous rash, in keeping with the Holster sign. (d) CT imaging on initial presentation showing mild left lower lobe subpleural consolidation and right lower lobe patchy consolidation (e) Interval CT imaging 6 weeks later showing rapidly progressive ground-glass changes and pneumomediastinum.

On examination, she had mechanic’s hands, peri-ungual erythema, Gottron’s papules and a diffuse violaceous rash on the lateral aspect of her thighs (Fig. 1). There was clinically evident arthritis affecting her wrists, knees, ankles and small joints of the fingers. Power was 5/5 in all limb movements. Results and management are summarised in Table 1.

**Table 1:** Table demonstrating timeline of events throughout diagnosis and management of case A

Time	Clinical Progress	Pertinent Examination Findings	Pertinent Results	Management
0	Reviewed in Same Day Emergency Care with a maculopapular rash on the thighs, erythema and a burning sensation on the fingertips, joint swelling and a dry cough.			
1 day	Rheumatology and dermatology review. DM without evident muscle involvement was suspected.	Peri-ungual swelling and erythema, clinically evident arthritis of multiple joints, mechanic’s hands and bibasal lung crepitations.	CRP 2.2 mg/L (<5 mg/L) CK 133 U/L (40-320 U/L)	Admission under the medical team for investigation of suspected DM.
2 days			CT TAP: Mild bibasal consolidation, small volume mediastinal and hilar lymphadenopathy (likely reactive).	
4 days			MRI thighs: Mild oedema at the iliopsoas myotendinous junctions and within the proximal lateral gastrocnemius muscles bilaterally. EMG/NCS: Normal	
6days			ANA weakly positive (1:80, speckled pattern) ENA, ANCA, dsDNA negative	Discharged home at patients request with urgent outpatient follow-up and PET scan.
8 days			PET scan: FDG avid arthropathy and subpleural ground-glass opacification and consolidation in both lungs. Low-grade uptake in mediastinal, hilar, axillary and groin lymph nodes.	
2 weeks	Seen in rheumatology clinic. Ongoing joint pain, swelling and stiffness.			Prednisolone 40 mg daily weaning course.
4 weeks	Seen in medical day unit for IV methylprednisolone.			1x500mg IV methylprednisolone Methotrexate 15 mg weekly.
5 weeks, 5 days	ED presentation with cough, yellow sputum, fevers, breathlessness.	Sats 94% RA. Crackles right chest base. Isolated low-grade fever (37.6°C).	CRP 17 (<5 mg/L)	Oral clarithromycin 500 mg BD.
6 weeks	Re-presented to ED with worsening cough and shortness of breath.	Sats 93% on 10 L supplemental oxygen. Diffuse bibasal crepitations.	CRP 37 mg/L (<5 mg/L) Lymphocytes 0.3x10^9^/L (1.0–4.0x10^9^/L)	Admission under the general medical team. Commenced on IV Clarithromycin 500 mg BD and IV Teicoplanin 800 mg OD. Dose of prednisolone doubled to 60 mg.
6 weeks, 2 days			ABG pO2 12.5, pCO2 4.6 (10 L supplemental oxygen). HRCT: Widespread ground-glass changes.	Transferred to ICU for respiratory support.
7 weeks	Ongoing desaturations requiring Optiflow. Discussed with ILD team at a tertiary centre. MDA-5-positive rapidly progressive ILD was suspected. ECMO retrieved the same day.	Sats 94% on Optiflow (40 l/min FiO2 50%).	CRP 44 (<5 mg/L) Procalcitonin 0.21 (<0.5ug/L) CTPA: Extensive ground-glass changes and consolidation with significant progression since the last scan (5 days ago) and pneumomediastinum.	IV methylprednisolone 500 mg x 1 At tertiary centre IV methylprednisolone 1000 mg x 3 Rituximab 1 g x 2 Cyclophosphamide 800 mg & 1000 mg Tofacitinib 5 mg BD Tacrolimus Dose guided by trough levels
8 weeks			MDA-5 and Ro-52 Antibody Positive	
4 months	Patient died.			

Abbreviations: ABG—Arterial Blood Gas, ANA—Anti-Nuclear Antibodies, ANCA—Antineutrophilic Cytoplasmic Antibody, BD—Twice Daily, CK—Creatine Kinase, CRP—C Reactive Protein, CTPA—CT Pulmonary Angiogram, CT TAP—CT Thorax-Abdomen-Pelvis, DM—Dermatomyositis, dsDNA—Double Stranded DNA, ECMO—Extra Corporeal Membrane Oxygenation, ED—Emergency Department, EMG—Electromyography, ENA—Extractable Nuclear Antigen, FDG—Fluorodeoxyglucose, FiO2—Fraction of Inspired Oxygen, Hb—Haemoglobin, HRCT—High Resolution CT, ICU—Intensive Care Unit, ILD—Interstitial Lung Disease, IV—Intravenous, MRI—Magnetic Resonance Imaging, NCS—Nerve Conduction Studies, PET—Positron Emission Tomography, RA—Room Air, RP-ILD—Rapidly Progressive ILD, Sats—Saturations.

Creatine kinase (CK) and inflammatory markers were normal. A CT thorax-abdomen-pelvis (TAP) revealed no underlying malignancy and MRI thighs showed no evidence of myositis. PET-CT demonstrated a symmetrical inflammatory polyarthropathy and features of an organising pneumonia. A skin biopsy from the thigh revealed subtle non-specific chronic inflammatory changes, and direct immunofluorescence was negative for IgG, IgM, IgA and C3. A systemic autoimmune condition was suspected, and over the ensuing month, she received methotrexate (15 mg weekly) and corticosteroids (oral prednisolone with a dose ranging between 30 mg to 60 mg and a single intravenous (IV) dose of methylprednisolone 500 mg) for treatment of the inflammatory arthritis, whilst awaiting the results from the MSA panel.

**Figure 2:**
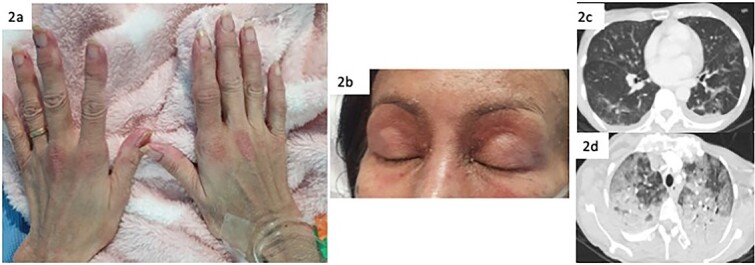
Images of the skin and CT changes seen in case B (a) Dorsum of the hands showing erythematous scaly papules on the metacarpophalangeal joints, consistent with Gottron’s papules (b) Anterior view of the face with mild erythema and scale in the periorbital regions and anterior scalp, with subtle purplish discolouration on the eyelids, in keeping with heliotrope rash. (c) CT imaging on initial presentation showing consolidation in both lungs with scattered nodularity and ground-glass changes (d) Interval CT imaging one week later showing rapidly progressive ground-glass changes with ‘crazy paving’ pattern and extensive bilateral consolidation.

Six weeks after presentation, she attended the Emergency Department (ED) due to progressive respiratory symptoms and was discharged with clarithromycin for suspected community-acquired pneumonia. She re-presented two days later and was found to be in type 1 respiratory failure (T1RF). She was admitted and IV teicoplanin, clarithromycin and Tamiflu were commenced, for the management of suspected infection. High-Resolution CT (HRCT) Thorax showed widespread ground-glass densities, bibasal consolidation, pneumothoraces and pneumomediastinum (Fig. 2), a dramatic change from the previous CT. She was transferred to the intensive care unit (ICU) for high-flow oxygen and referred to a tertiary centre for discussion at the ILD MDT. Anti-MDA-5 DM was suspected and she was transferred for urgent extra-corporeal membrane oxygenation (ECMO). Aggressive immunosuppression was commenced, including IV methylprednisolone (three 1 g pulses followed by weaning doses of oral prednisolone), tofacitinib 5 mg twice daily, tacrolimus (dose guided by trough levels), three pulses of IV cyclophosphamide (800 mg–1000 mg) and IV rituximab (2 doses of 1 g).

Strong positive anti-MDA-5 and Ro-52 positivity was later confirmed using myositis immunoblot. She died 2 months later, due to respiratory failure and progressive treatment-resistant pneumomediastinum.

### Case B

A 60 year-old female of Chinese heritage and no past medical history, presented to ophthalmology with left eyelid swelling and was diagnosed with pre-septal cellulitis. Six weeks later, she presented to ED with fatigue, reduced mobility and 10 kg unintentional weight loss. Examination revealed cachexia and ulcerating lesions on her shoulders and hips, thought to be pressure sores. Initial investigations showed normocytic anaemia, hypoalbuminaemia and raised C Reactive Protein (CRP) (Table 2). Malignancy was suspected and she was admitted for investigations.

**Table 2:** Table demonstrating timeline of events throughout diagnosis and management of case B

Time	Clinical Progress	Pertinent Examination Findings	Pertinent Results	Management
0	Diagnosis of preseptal cellulitis by ophthalmology.	Left eyelid swelling.		Oral co-amoxiclav.
2 weeks	Reviewed in Same Day Emergency Care with palpitations, fatigue and fevers.		ECG: Normal sinus rhythm	Discharge with plan for outpatient 24-hour tape.
6 weeks	Presentation to ED with fatigue, 10 kg unintentional weight loss and ulcerating skin sores.	Hypotension and tachycardia. Ulcerating lesions to shoulders and sacrum.	Bloods: Hb 99 g/L (120–150 g/L) WBC 4.8x10^9^/L (3.6-11x10^9^/L) CRP 19 mg/L (<5 mg/L) Albumin 18 g/L (25-50 g/L) ECG: Sinus tachycardia CXR: Mild pulmonary congestion	Admission under the medical team for investigation of suspected underlying malignancy, dietician input and safeguarding review (for presumed sacral pressure sores).
6–7 weeks	Transferred to care of the elderly ward. Neurology advice: EMG/NCS, MRI head and C-spine, and anti-neuronal antibodies. Dermatology review: Skin changes mild and non-specific but differential diagnosis included dermatomyositis, skin biopsy performed and rheumatology review advised. Rheumatology advice: Autoantibody screen including MSA, await NCS/EMG and MRI thighs +/− biopsy.	Fasciculations, no clinical evidence of proximal myopathy. Gottron’s papules.	Bloods: CRP 23 mg/L to 104 mg/L (<5 mg/L) CK 1098 U/L (40-320 U/L) Ferritin 3200 ng/ml (41-440 ng/ml) LDH 858 U/L (<250 U/L) Skin biopsy: Features in keeping with an immune vasculitis. CT Thorax-Abdomen-Pelvis: No evidence for malignancy, but patches of collapse/consolidation in both lungs with scattered nodularity. MRI head: Small cavernoma in the right pons (likely incidental). MRI spine: Degenerative changes. NCS/EMG: Predominantly proximal myopathic changes. MRI calves: Diffuse oedema throughout posterior calf musculature in keeping with myositis.	
7.5 weeks	New oxygen requirement, treated for presumed hospital acquired pneumonia.	Isolated low-grade fever (37.7°C)	CRP 104 mg/L (<5 mg/L) Procalcitonin 0.68ug/L (<0.5ug/L)	Piperacillin-tazobactam.
8 weeks	Worsening type 1 respiratory failure.		ABG on 15 L NRB mask: pH 7.49, pO2 33.4, pCO2 4.9. CTPA: Progressive bilateral consolidation, widespread nodular and ground-glass changes, increasing bilateral effusions.	Transferred to the respiratory ward for Optiflow.
8 weeks, 1 day			PJP swab positive.	Transferred to ICU for respiratory support. Co-trimoxazole added.
8 weeks, 5 days	Dermatology and rheumatology discussion: Likely anti-MDA-5 DM with RP-ILD. Discussed with tertiary centre and ECMO retrieved within 3 hours of discussion.	Dermatology consultant review: Further skin signs included mechanic’s hands, subtle Gottron’s papules, left elbow skin ulceration and nail-fold microhaemorrhages consistent with DM.		IV methylprednisolone 500 mg x 1 At tertiary centre: IV methylprednisolone 1000 mg x 2 Rituximab 1 g x 2 Cyclophosphamide 500 mg x 2 Tofacitinib 5 mg BD Tacrolimus Dose guided by trough levels
10 weeks			MDA-5 and Ro-52 Antibody positive	
11.5 weeks	Patient died			

Abbreviations: ABG—Arterial Blood Gas, BD—Twice Daily, CK—Creatine Kinase, CRP—C Reactive Protein, C-spine—Cervical-Spine, CTPA—CT Pulmonary Angiogram, CXR—Chest X ray, DM—Dermatomyositis, ECG—Electrocardiogram, ECMO—Extra Corporeal Membrane Oxygenation, ED—Emergency Department, EMG—Electromyography, Hb—Haemoglobin, HRCT—High Resolution CT, ICU—Intensive Care Unit, ILD—Interstitial Lung Disease, IV—Intravenous, LDH—Lactate Dehydrogenase, MRI—Magnetic Resonance Imaging, MSA—Myositis Specific Antibodies, NCS—Nerve Conduction Studies, NRB—Non-Rebreather, PJP—Pneumocystis Jirovecii Pneumonia, RP-ILD—Rapidly Progressive ILD, Sats—Saturations, WBC—White blood cells.

A CT TAP showed patches of consolidation, nodularity and ground-glass changes in both lungs, with no evidence of malignancy (Fig. 2).

Fasciculations were noted in her tongue and upper limbs, with normal power on initial assessment. A diagnosis of myositis was supported by an elevated CK (1098 U/L), myopathic changes on electromyography (EMG) and MRI lower limbs demonstrating diffuse oedema.

Non-specific erythema and scale on the face and dorsal aspects of the hands and fingers were noted, raising suspicion for Gottron’s papules. Skin biopsy from an erythematous papule on the right third metacarpophalangeal joint showed epidermal basal vacuolation with colloid bodies, and vascular endothelial swelling with fibrinoid necrosis and neutrophilic migration into the vessel wall. Direct immunofluorescence showed linear basement membrane staining with IgA, and positive staining of vessel walls with IgM and C3. The features were suggestive of an immune vasculitic process, which was not felt to be classical for DM. She later developed skin ulceration on the left elbow, mechanic’s hands, and nailfold microhaemorrhages.

On day 11, she desaturated and was commenced on piperacillin-tazobactam for presumed hospital-acquired-pneumonia. Four days later, she was transferred to ICU for the management of severe T1RF. A repeat CT thorax showed worsening ground-glass changes, bilateral consolidation and moderate effusions (Fig. 4).

Given her skin changes, investigations consistent with myositis and rapidly progressive respiratory failure, anti-MDA-5 DM was suspected. She was referred to a tertiary centre for ECMO and aggressive immunosuppression, including IV methylprednisolone (two 1 g pulses followed by weaning doses of oral prednisolone), tofacitinib 5 mg twice daily, tacrolimus (dose guided by levels), three pulses of IV cyclophosphamide (2 doses 500 mg) and IV rituximab (2 doses of 1 g).

Immunology confirmed strong positive anti-MDA-5 and Ro-52 autoantibodies on myositis immunoblot. She died five weeks after admission.

## Discussion

Anti-MDA-5 DM is a multi-system condition that can present with cutaneous, muscular, articular and respiratory involvement. Cutaneous features include classical DM signs; the heliotrope sign, shawl sign, Gottron’s papules and mechanic’s hands, which may not always be present. Deep painful skin ulcers and palmar papules (also known as inverse Gottron’s papules) are more specific to anti-MDA-5 DM^5^. Histology of DM skin typically shows an interface dermatitis, and in the MDA-5 subtype there may be epidermal necrosis and vasculopathy^5^, but the findings can be non-specific. Both of our patients presented with cutaneous stigmata of DM. Patient A had Gottron’s papules, mechanics hands and nailfold changes, whilst the rash on her thighs likely represented the Holster sign. Patient B presented with deep skin ulcerations, initially misdiagnosed as pressure sores, and with periorbital oedema, thought to be pre-septal'cellulitis.

Like case A, anti-MDA-5 DM is classically associated with CADM. Cohort studies estimate that 23%–100% of CADM cases test positive for anti-MDA-5 antibodies [6]. Other clinical manifestations include inflammatory arthritis and constitutional features e.g. fevers and fatigue [6].

ILD is a common manifestation of anti-MDA-5 DM, occurring in 82%–100% of East-Asian cohorts, and 38%–73% of Caucasian patients [6]. It is vital to identify RP-ILD promptly, due to its associated mortality rates, which are estimated to be greater than 50% [6]. The diagnosis of RP-ILD can be challenging and in both of our cases, it was initially misdiagnosed as infection and/or fluid overload. Unfortunately, due to the aggressive nature of anti-MDA-5 DM, both patients died from respiratory failure.

General Practitioners and General Medicine Physicians must be aware of the clinical features of DM, including skin rashes, for swift onwards rheumatology referral. Early and routine testing for MSAs is vital in the diagnostic work-up of DM and MSA subtypes characterise distinct clinical phenotypes including risk for ILD and cancer [7]. When anti-MDA-5 antibodies are detected, clinicians should be alert to the risk of RP-ILD and promptly request appropriate investigations, including HRCT and lung function testing.

Anti-MDA-5 DM is a complex and heterogenous condition, with varied clinical presentations and outcomes. A French cohort study of 83 patients identified three distinct clinical phenotypes of anti-MDA-5 DM [7]. In similarity to our cases, the first subgroup corresponded to patients with a high prevalence of RP-ILD and a poor prognosis. In some of these patients, a hyperinflammatory state with persistent fevers and hyperferritinaemia was seen [3]. The second subgroup included patients with predominant skin and muscle involvement and a good prognosis. The final subgroup included those with severe skin vasculopathy and clinical myositis. These patients were predominantly male and had an intermediate prognosis.

Given the clinical heterogeneity of anti-MDA-5 DM, it is important to identify those at risk of rapid disease progression, who should be targeted for early aggressive immunomodulatory therapies. Predictors of poor prognosis and a higher risk of mortality include clinical factors such as older age, male gender, persistent fevers and oxygen desaturations [8], and laboratory features including elevated ferritin, CRP, lactate dehydrogenase and lymphopenia [3]. Some studies suggest that the co-existence of anti-Ro-52 antibodies is higher in patients with anti-MDA-5 DM and RP-ILD [9].

In conclusion, we present two cases of anti-MDA-5 DM with RP-ILD. These cases highlight the challenges associated with diagnosis, due to heterogeneity in clinical presentation, and the potential for rapid respiratory deterioration.

## Conflict of interest statement

The authors have no conflicts of interest to declare.

## Consent

Informed written consent was taken from both patients’ next of kin.
